# Predicting the Magnet Strength Using a Tablet-Based Tool for Patients Undergoing Cochlear Implantation

**DOI:** 10.7759/cureus.46417

**Published:** 2023-10-03

**Authors:** Mohamed Garrada, Aseel Baflah, Faisal Zawawi

**Affiliations:** 1 Otolaryngology and Head & Neck surgery, King Abdulaziz University, Jeddah, SAU; 2 Otolaryngology and Head & Neck Surgery, King Abdulaziz University, Jeddah, SAU

**Keywords:** otoplan, cochlear implants < audiology, magnet, hearing screening, cochlear implants

## Abstract

Objectives

Preoperative image analysis of skin flap thickness and determining the required magnet strength are important in the management of CI surgery. The primary aim of this study is to analyze the application of OTOPLAN®, a tablet-based otological preplanning tool, in assessing skin flap thickness. The secondary aim was to determine if there is any correlation between the skin flap thickness and the selected magnet strength.

Methods

Fifty-seven computer tomography (CT) image datasets of temporal bones of cochlear implant (CI) patients were collected. CE marked OTOPLAN^®^ planning otology software was used to load the patient’s preoperative images for measuring the skin flap thickness in both axial and coronal views. To standardize the skin flap thickness measurement, the top of the pinna on the side of implantation was taken as the measurement point.

Results

The mean age of the patients was 7.98 ± 1.54 years. The body mass index (BMI) was not considered in this study. The average skin flap thickness was 4.5 ± 1.2 mm (range: 2-7 mm). The inter-rater reliability test revealed strong agreement between the two reviewers (Cronbach’s alpha = 0.90). The majority of the patients were fitted with a magnet strength of 3. A statistically significant positive correlation was observed between the skin flap thickness and the age of the patients (r = 0.69, p = 0.002). Also, between the skin flap thickness and the magnet strength, a strong positive correlation was observed (r = 0.82, p < 0.0001).

Conclusions

OTOPLAN® is a reliable tool in the measurement of skin flap thickness with little effort. The age and the magnet strength were positively correlated with the skin flap thickness.

## Introduction

Cochlear implantation is the state-of-the-art treatment option for sensorineural hearing loss conditions [1]. The cochlear implant (CI) device comprises an external audio processor with an inbuilt microphone to capture the sound signal. The audio processor converts the sound signal into detailed digital signals, applying signal processing algorithms, and transmits those to the implantable electronics via an inductive link. The implant electronics then convert these digital signals to electrical impulses and pass them to the inner ear through the intracochlear electrode array. The inductive link coil of the external component is held on the surface of the skin flap over the implantable components by magnetic attraction between the external and the implantable components [2].

The importance of the success of the CI depends on the surgery mostly, but overtime, the procedure is getting simplified and easier. The magnet used in the CI device should possess optimal magnetic strength in order to maintain the integrity of the skin overlying the implant and, at the same time, be strong enough to hold the inductive link coil of the external component over the implantable part [3,4]. The implantable part always comes with a standard magnet, whereas the coil of the external component can be equipped with the magnet of desired magnetic strength. Often, CI’s external processors are shipped to the clinic with a default magnetic strength, which may or may not fit the patient’s needs. This may pose problems to patients who may need strong or weak magnets in the inductive link coil. A strong magnet might be needed in patients with thick skin flaps, whereas a weak magnet is just enough in patients with thin skin flaps. 

Magnets with higher strength can result in many consequences, such as disrupting the skin, causing infection, and, in the worst-case scenario possible, implant extrusion leading to revision surgeries [5]. Moreover, a magnet with lower strength can lead to poor retention of the inductive link coil, and this may further lead to a higher power consumption from the battery [5]. Custom ordering the required magnet strength could delay the fitting of the audio processor, which further leads to an overall delay in restoring hearing.

The objective of this study is to propose a method for predicting the magnet strength preoperatively based on the skin flap thickness of the CI recipients.

## Materials and methods

Study design

This retrospective study was conducted in a tertiary academic referral center. The study was approved by the biomedical ethics research committee (Reference No. 587-21) as a non-interventional retrospective record review study. The collected data included age, magnet strength, and pre-operative imaging. All patients who underwent CI surgery with MED-EL devices with the same magnet design (implant types: C40+, Sonata, Concerto, and Synchrony) between 2015 and 2020 and who possessed high-resolution computed tomography (CT) images were included in this study. Patients with missing records and those who didn’t follow up for a long time were excluded.

Cochlear implant procedure

In our center, the minimally invasive CI procedure starts with a 3 cm long postauricular incision, followed by the elevation of the posterosuperior flap to create a pocket large enough to fit the receiver-stimulator. Mastoidectomy and a posterior tympanotomy technique were used, followed by receiver-stimulator bed excavation. The round window or a cochleostomy method was used to insert all electrode arrays in all patients, and the window or cochleostomy was sealed with temporalis fascia.

The periosteum pocket was made to ensure device fixation with no suturing for the internal receiver. The skin and subcutaneous tissue were closed in layers with very close sutures to complete the surgery. Intraoperative measurements were done for all patients to measure the auditory nerve response and to confirm the device's functionality. As a clinical routine in our clinic, the external devices are activated for all patients three weeks after surgery as a clinical routine.

Skin flap thickness measurement

**Figure 1:**
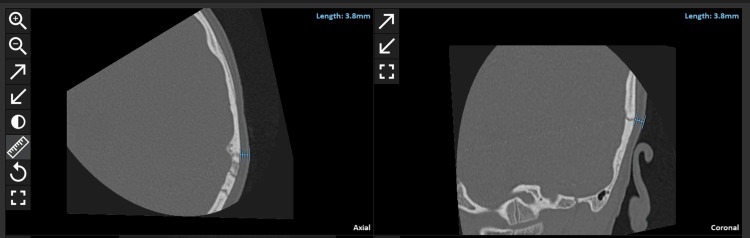
Measuring the skin thickness on the OTOPLAN software in coronal and axial views

The preoperative CT images were uploaded to the OTOPLAN® V.03 DICOM viewer and surgical planning software (CAScination AG, Bern, Switzerland). The pre-operative CT scans were uploaded to this software to measure the skin flap thickness simultaneously in both axial and coronal views. The measurements were done by two independent reviewers with the same level of experience in dealing with both inner ear anatomy and OTOPLAN. To standardize the skin flap thickness measurement, the top of the pinna on the side of implantation was taken as the measurement point (Figure 1). Both reviewers were blinded to the magnet strength fitted to the assessed patients.

Statistical analysis

The mean, standard deviation, and ranges of patients' descriptive demographic characteristics were computed (i.e., minimum and maximum values). Following confirmation of their normality, the Pearson correlation calculation was used to test the correlation between the data of each two specific columns. The statistical significance level was set at p = 0.05. GraphPad PrismTM version 8.4.0 was used for statistical analysis (GraphPad Software, La Jolla, CA, USA).

## Results

Fifty-seven ears (42 patients) that received MED-EL CI systems met the inclusion criteria of this study, and their CT scans were compatible with OTOPLAN® software. The medical records were reviewed and revealed that 41 ears were implanted on the right side (71.2%) and 17 were implanted on the left side (29.8%). All included patients had no incidence of device issues after CI, like infection, migration, or failure.

The mean age of the patients included in this study was 7.98 ± 9.8 years. The body mass index (BMI) was not considered in this study. The majority of patients (42.5%) were fitted with a magnet strength of 3, and the average skin flap thickness measured was 4.5 ± 1.2 mm (range: 2-7 mm). The inter-rater reliability test revealed perfect agreement between the two reviewers (Cronbach’s alpha = 0.90). Table 1 depicts the magnet strength used in patients of various age groups and skin flap thicknesses.

**Table 1:** Baseline data mean and standard deviation of the magnet strength, age of patients, and skin flap thickness

Magnet strength	Age (years; SD)	Skin flap thickness (mm; SD)
1	1.75 ± 0.96	3.12 ± 0.21
2	4.0 ± 2.0	3.45 ± 0.85
3	4.47 ± 0.68	4.47 ± 0.63
4	5.70 ± 0.97	5.62 ± 0.88
5	6.08 ± 0.66	6.30 ± 0.4

**Figure 2:**
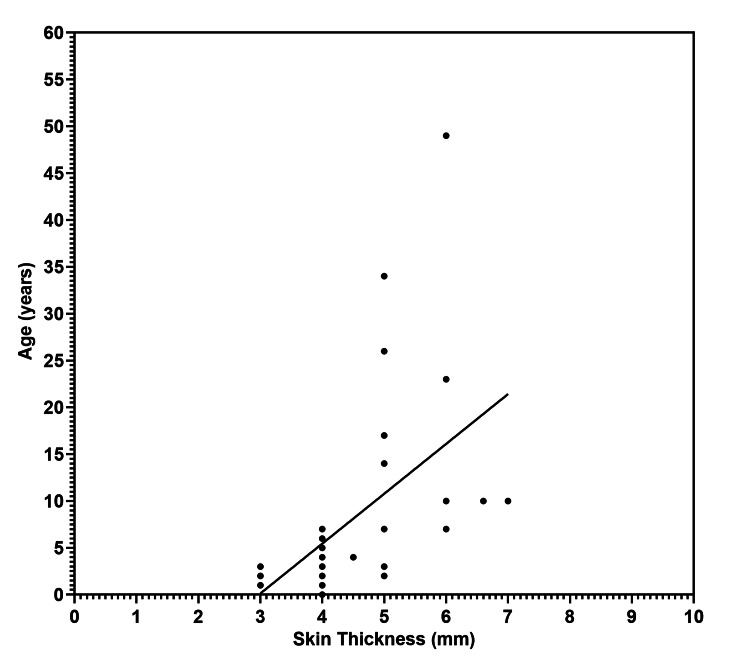
Scatter plot showing the significant positive relationship between the skin flap thickness (mm) and age of patients (years)

**Figure 3:**
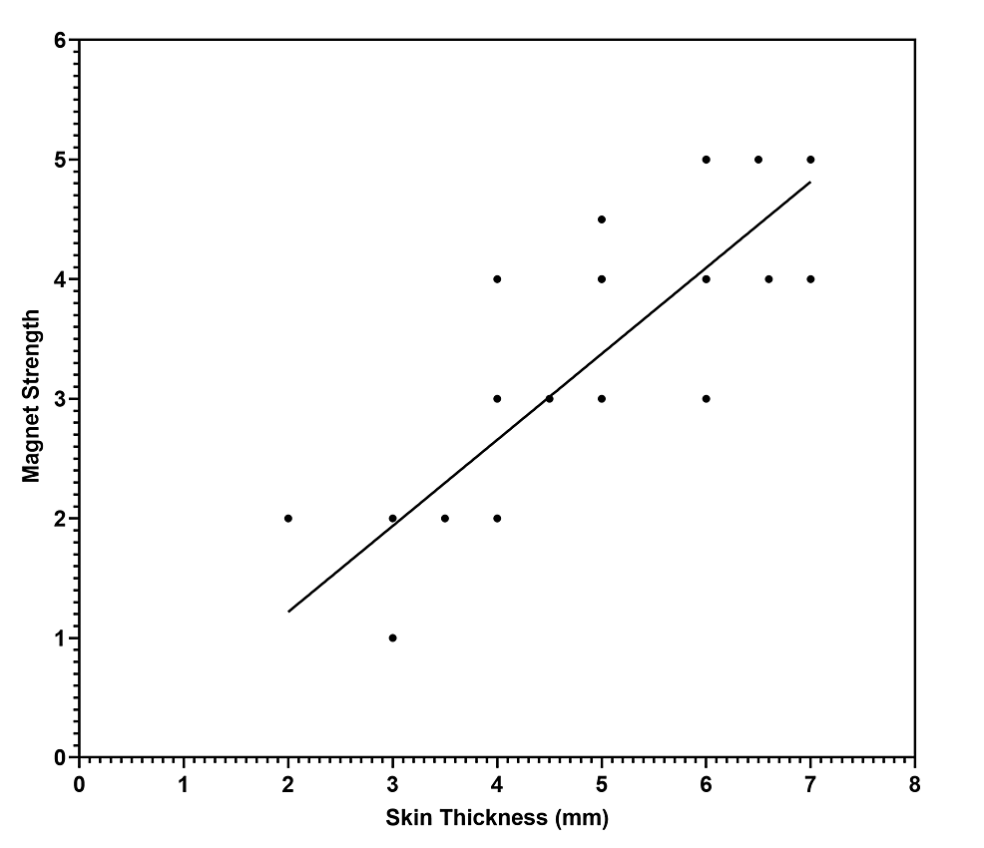
Scatter plot showing the significant positive correlation between the skin thickness (mm) and magnet strength

A statistically significant positive correlation was observed between the skin flap thickness and the age of the patients (r = 0.69, p = 0.002) (Figure 2). Also, as shown in Figure 3, there was a strong positive correlation between the skin flap thickness and the magnet strength, which was statistically significant (r = 0.82, p < 0.0001).

Moreover, the analysis resulted in a mathematical equation that can be used to predict the magnet strength based on the measured skin thickness: M = 0.72 * S − 0.22, where M indicates the magnet strength and S indicates the skin thickness in mm.

**Figure 4:**
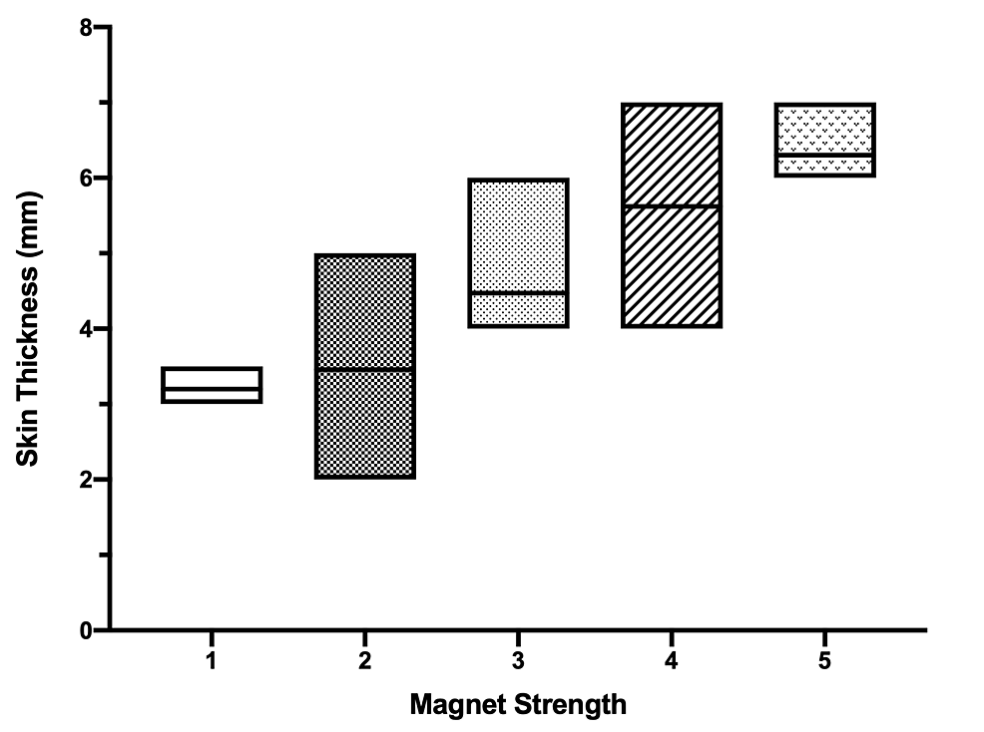
Boxplot represents the magnet strength in relation to skin thickness (mm)

Categories of magnet strength and their relevant skin flap thickness values are shown in Figure 4. As expected, higher magnet strength was used in patients with higher skin flap thickness.

## Discussion

This study investigated the relationship of skin flap thickness with the age of the patients and the magnet strength used in the inductive link coil of the external processor. The current findings within our population suggest that the magnet strength can be chosen based on the patient’s age and skin flap thickness. A future study with a larger patient size and diametrically polarized magnet could further support our findings. 

The majority of the patients in the current study were fitted with a magnet strength of between 2 and 4, which is highly consistent with the study of Searle et al. [5]. They reported that their findings were obtained by using devices from Cochlear®, whereas our study findings were obtained by using MED-EL devices. A magnet strength of 5 was used in patients whose skin flap thickness exceeded 6 mm, which is an indicator for our clinic to predict the magnet strength based on the skin flap thickness.

Choosing the higher magnet strength in cases with higher skin flap thickness is superior to surgically reducing the skin flap thickness, as it eliminates the issues of postoperative infection, necrosis, and abscess formation [6,7]. Also, the discomfort of wearing headbands can be eliminated if the proper magnet strength is chosen to match the skin flap thickness. Overall, choosing the optimal magnet strength would optimize the transfer of signal from the external component to the implantable component of the CI [8]. BMI was not considered in the current study, whereas Posner et al. [9] reported greater skin flap thickness with greater BMIs and recommended supra-fascial placement of the implant coil to avoid skin flap reduction surgery.

Almuhawas et al. [10] earlier reported an exponential increase in mastoid thickness until around 14 years of age, and our current finding of increasing skin flap thickness with the age of the patient conveys that there is an overall anatomical growth with the age of the patient. Sharma et al. [11] earlier reported a strong correlation between age and scalp thickness, which is in line with our findings. The skin flap thickness increasing with age would mean that there is a necessity to consider changing the magnet strength in the inductive coil of the external processor as needed overtime to have the effective transfer of signal. 

The reliable measurement of the skin flap thickness depends on consistent identification of the anatomical landmarks in the CT scans of patients with differing image orientations. Rees et al. [12] earlier compared needle piercing with imaging techniques to measure skin flap thickness by applying ultrasound, CT, and magnetic resonance imaging (MRI) and concluded that ultrasound/CT had a higher degree of accuracy compared to needle/CT and needle/MRI. OTOPLAN® software enables visualizing the anatomical landmark simultaneously in both axial and coronal planes, validating the measurement in situ. In the current study, our patient population was implanted with different implant types, including the one that has a diametric magnet design from a single CI manufacturer. Yet, a strong positive correlation was observed between the skin flap thickness and the magnet strength. A similar finding was observed in Adkins et al. [13] and Searle et al. [5] using the Cochlear® CI devices. Nowadays, all CI manufacturers are offering the diametric magnet design in their implants, and the future study would focus on increasing the CI patient population implanted with diametric magnet design from all three major CI manufacturers. This study has some limitations, such as the sample size and the retrospective design.

## Conclusions

OTOPLAN® is a reliable tool for measuring the skin flap thickness with little effort, which can be used to predict the required magnet strength. The age of the patients is positively correlated to the skin flap thickness and the magnet strength used in the inductive coil of the external processor. Often, the CI companies supply the audio processor with the standard magnet strength, predicting that the magnet strength from the pre-operative image analysis would help the patient receive the desired magnet strength on time and start using the audio processor soon after the CI surgery, potentially minimizing skin-related issues.
